# Serum parameters in the spectrum of coeliac disease: beyond standard antibody testing - a cohort study

**DOI:** 10.1186/1471-230X-12-159

**Published:** 2012-11-12

**Authors:** Greetje J Tack, Roy L J van Wanrooij, B Mary E Von Blomberg, Hedayat Amini, Veerle M H Coupe, Petra Bonnet, Chris J J Mulder, Marco W J Schreurs

**Affiliations:** 1 Department of Gastroenterology and Hepatology, VU University Medical Centre, PO Box 7057, Amsterdam 1007 MB, The Netherlands; 2 Department of Pathology, VU University Medical Centre, Amsterdam The Netherlands; 3 Department of Epidemiology and Biostatistics, VU University Medical Centre, Amsterdam The Netherlands; 4 Department of Immunology, Erasmus MC, University Medical Centre, Rotterdam, The Netherlands

**Keywords:** Coeliac disease, Refractory coeliac disease, Enteropathy associated T-cell lymphoma, Cytokines, Immunological and biochemical parameters

## Abstract

**Background:**

Invasive techniques are still required to distinguish between uncomplicated and complicated forms of CD.

**Methods:**

We set out to investigate the potential use of novel serum parameters, including IL-6, IL-8, IL-17, IL-22, sCD25, sCD27, granzyme-B, sMICA and sCTLA-4 in patients diagnosed with active CD, CD on a GFD, Refractory coeliac disease (RCD) type I and II, and enteropathy associated T-cell lymphoma (EATL).

**Results:**

In both active CD and RCDI-II elevated levels of the proinflammatory IL-8, IL-17 and sCD25 were observed. In addition, RCDII patients displayed higher serum levels of soluble granzyme-B and IL-6 in comparison to active CD patients. In contrast, no differences between RCDI and active CD or RCDII were observed. Furthermore, EATL patients displayed higher levels of IL-6 as compared to all other groups.

**Conclusions:**

A series of novel serum parameters reveal distinctive immunological characteristics of RCDII and EATL in comparison to uncomplicated CD and RCDI.

## Background

Coeliac disease (CD) is a chronic immune-mediated inflammation of the small intestine caused by a permanent state of intolerance to ingested gluten proteins affecting genetically susceptible individuals. Its hallmarks, lymphocytic infiltration of the lamina propria, expansion of the intraepithelial lymphocyte (IEL) population, hyperplasia of the crypts and atrophy of the villi are mediated by the interplay between an innate and adaptive immune response against gluten [1]. The complex of deamidated gliadin peptide interacting with the HLA-DQ2 and/or –DQ8 heterodimers on antigen presenting cells, is capable of activating the lamina propria T-helper lymphocytes, thereby initiating antibody production and a gluten-specific pro-inflammatory type 1 T-cell response (Th1) [1]. Th1 cells play an important role in pathogenesis of CD by secreting interleukin-2 (IL-2) that induces proliferation of T-lymphocytes and, in particular, by secreting the pro-inflammatory cytokine interferon-gamma (IFN-γ) [2]. Furthermore, recent evidence has indicated that Th17 cells play a pathogenic role in CD [3-5]. In addition, interleukin-15 (IL-15) activates a cytotoxic response of intra-epithelial lymphocytes through IFN-y release and upregulation of NKG2D. In combination with epithelial stress, the NKG2D ligand MHC Class I chain-related A (MICA) is upregulated by enterocytes [6,7]. The interaction of MICA and NKG2D induces enterocyte destruction by IELs.

A gluten-free diet (GFD) results in mucosal recovery in the majority of patients, who are referred to as uncomplicated CD-patients. However, a small subset of adult onset CD patients fails to regain intestinal homeostasis after elimination of dietary gluten, or symptoms recur after initial response [8]. After careful evaluation of dietary compliance and exclusion of other possible disease-entities causing villous atrophy, these patients are diagnosed to suffer from refractory coeliac disease (RCD) [9]. RCD is considered a complicated form of CD, and is divided into type I (RCDI), when patients lack an aberrant IEL population, or type II (RCDII) in which a substantial (>20%) aberrant IEL population is found in the small intestinal mucosa [10,11]. An aggressive type of lymphoma which carries a dismal prognosis, the enteropathy-associated T-cell lymphoma (EATL), is thought to arise from this aberrant IEL population. An interesting observation is that both aberrant IELs as well as EATL cells display a cytotoxic phenotype and contain high levels of granzyme-B [12,13], which could therefore serve as a marker for complicated CD.

In consequence of a good response to immunosuppressive therapy, RCDI patients have a better prognosis than RCDII patients [14-17]. Therefore, early identification of CD patients developing RCDII and/or EATL enables early intervention, which will likely reduce morbidity and mortality.

Currently, antibodies against tissue transglutaminase (TGA), anti-endomysium (EMA) and deamidated gliadin peptides (DGPA) provide valuable and generally accepted serum parameters for the diagnosis and follow-up of uncomplicated CD. However, these antibodies are of no use in predicting and monitoring both types of RCD and EATL, implying that histological and flow cytometric analysis of duodenal biopsies are still required to distinguish between the uncomplicated and complicated forms of CD. Additional serum markers could potentially provide us with a minimal-invasive, easy applicable test without the need to perform a gastro-duodenal endoscopy. In addition, immunological markers in the peripheral blood could provide more insight in the similarities and differences of the pathophysiology underlying the CD spectrum.

Therefore, in the present study we evaluated several immunological and biochemical parameters in sera from the five stages of CD, including active CD (ACD), CD on GFD, RCDI-II and EATL, for their ability to differentiate between complicated and uncomplicated forms, and secondly, to gain insight in the pathophysiological relations between these disease entities. For this purpose, we analysed serum levels of the inflammatory cytokines IL-6, IL-8, IL-17and IL-22, the T-cell activation factors soluble (s)CD25 (IL2R-alpha) and sCD27, the T-cell dysregulation factor sCTLA-4, shown previously to be up-regulated in different autoimmune diseases, the cytotoxic T-cell parameter granzyme-B, and sMICA, previously shown to be associated with the presence of epithelial stress and malignancies.

## Methods

A retrospective cohort study was conducted at a tertiary referral centre for coeliac disease in The Netherlands. Patients previously diagnosed with (complicated) CD in the VU medical center were identified and included in our study when stored serum samples at time of diagnosis were available. Overall, 92 blood samples collected for diagnostic purposes between 1997 and 2010 were included. Serum levels of a substantial number of immunological markers were determined in the five different subsets of CD. In addition, results from several biochemical parameters of this cohort at time of diagnosis were collected from the electronic patient file in our centre.

### Patients

CD diagnosis was based on the ESPGHAN guidelines [18]. All patients included in the active CD group had positive EMA and/or TGA, and histological abnormalities grade III according to the modified Marsh classification consisting of intra-epithelial lymphocytosis, crypt hyperplasia and some degree of villous atrophy [19]. Furthermore, serum samples of these patients at time of an inactive phase of CD were collected. Remission of disease (GFD group) was defined by the disappearance of one or both CD antibodies upon a GFD, and if a gastro-duodenal endoscopy with subsequent collection of biopsies was performed during follow-up, normalisation of mucosal abnormalities (Marsh 0 or I) was required. Patients included in the RCD group had persisting or recurring symptoms and small intestinal villous atrophy, despite strict adherence to a GFD for at least one year (assessed by a dietitian and negative TGA/EMA). The clinically validated cut-off value of more than 20% of the IELs expressing an aberrant phenotype (surface CD3^-^, but cytoplasmic CD3^+^) as detected by flow cytometric analysis was used to distinguish RCD type I and II [10].

In total, 26 paired serum samples of CD patients at time of disease activity (ACD group) and after normalisation of the CD associated antibodies upon a GFD (GFD group), and of an additional 40 patients with complicated CD at diagnosis were included. The latter group consists of 12 RCDI, 16 RCDII and 12 EATL patients.

All procedures were in accordance with the regulations of the medical ethics committee, and all patients declared their informed consent to store and use their blood samples collected for regular diagnostics for further research.

### Serum parameters: Enzyme linked immunosorbent assay (ELISA)

Levels of cytokines IL-6, IL-8, IL-17 and IL-22 were determined in serum using commercial ELISA kits (Pelikine-compact™, Sanquin, Amsterdam, The Netherlands), according to the manufacturer’s instructions. Levels of soluble CD25 (sCD25), soluble CD27 (sCD27), soluble CTLA-4 (sCTLA-4), soluble MICA (sMICA) and granzyme-B were determined in serum using a specific commercial ELISA kit (Diaclone, Besancon, Cedex, France), according to the manufacturer’s instructions.

### Biochemical parameters

The concentration of C-reactive protein (CRP; g/L), erythrocyte sedimentation rate (ESR; mm per 1 h), leukocyte count (WBC; 10E9/L), albumin (g/L) and haemoglobin (Hb; mmol/L) were extracted from the hospital patient file for all patients included at time of diagnosis.

### Statistical analysis

Data were analysed and plotted using SPSS software (SPSS Inc. Chicago, Illinois, USA), using non-parametric tests as most variables examined in this study did not appear to be normally distributed. A Wilcoxon signed-rank test was used for pairwise comparison of the variability of immunological and biochemical parameters among the ACD and GFD group. The latter groups were individually compared to the complicated forms of CD by using the Kruskal-Wallis non-parametric test to identify possible serum and biochemical differences in the spectrum of CD. A receiver operating characteristic (ROC) curve was made of all significantly different parameters to represent the trade off between the false negative and false positive rates. As a considerable number of markers were determined, the level of significance was set at highly significant (p < 0.001) and moderately significant (0.001 < p <0.05).

## Results

**Figure 1:**
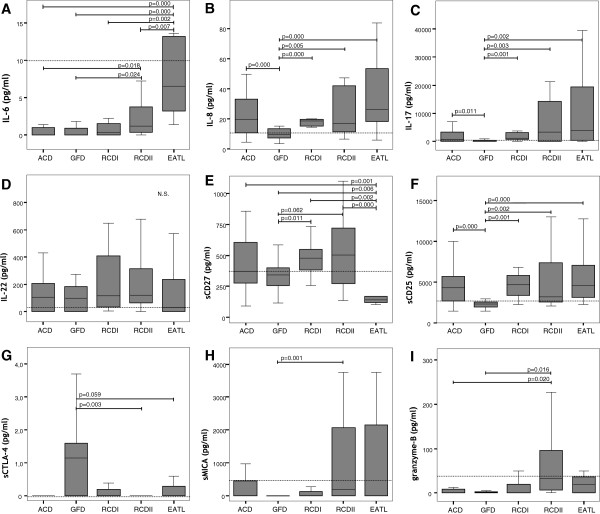
**A-I: Concentration of serum parameters in the different subsets of the CD spectrum: IL-6 (A) ; IL-8 (B); IL-17 (C); IL-22 (D); sCD25 (E); sCD27 (F) ; sMICA (G); sCTLA-4 (H); granzyme-B (I). **The dotted line corresponds with the upper level of the normal range. Abbreviations: CD, coeliac disease; ACD, active coeliac disease; GFD, response upon a gluten-free diet; RCD I, refractory coeliac disease type I; RCD II, refractory coeliac disease type II; EATL, enteropathy associated T-cell lymphoma.

Table 1 shows the characteristics of the five subsets of CD. Serum samples of 26 ACD patients at time of diagnosis and after remission of disease on a GFD were included. In all patients CD associated antibodies reverted to negative upon a GFD, and in 62% (16/26) a biopsy was taken which revealed mucosal healing (Marsh 0/I) in all those evaluated. Overall, 12 RCDI, 16 RCDII and 12 EATL patients were included. Significant differences were found for cytokine profiles between the five subsets of CD, as described in more detail below (Figure 1A-I).

**Table 1:** Characteristics of the different groups in the spectrum of coeliac disease

		**SPECTRUM OF CD**				
		**ACD**	**GFD**	**RCDI**	**RCDII**	**EATL**
		**(n = 26)**	**(n = 26)**	**(n = 12)**	**(n = 16)**	**(n = 12)**
**Age at CD** (**yr**)	Median, (SD;range)	42.5	45	50.5	61.5	61.5
		(16; 21-76)	( 15; 21-77)	(16; 19-69)	(13; 27-74)	(7; 48-66)
**Age at RCD**	Median, (SD;range)	-	-	59	65	64
				(11.5; 35-74)	(10; 47-77)	(8; 51-72)
**Age at EATL**	Median, (SD;range)	-	-	-	-	65
						(6; 51-74)
**HLA**-**staus**	DQ2 heterozygous	10	7	8	7	3
	DQ8 heterozygous	9	13			3
	DQ2/8 heterozygous	3	2	1	3	2
	DQ2 homozygous	4	4	3	6	4
**Marsh**	0		12			2
	I		4			
	II					1
	IIIA	11		8	9	5
	IIIB	5		3	4	3
	IIIC	10		1	3	1
	ND		10			
**EMA**	Negative (-)		26	12	15	7
	Dubious (+/-)				1	2
	Weak positive (+)	3				
	Positive (++)	11				0
	Stronglypositive(+++)	11				2
	ND	1				1
**TGA**	Negative		7	12	14	6
	Dubious				2	2
	Weak positive	8				1
	Positive	6				1
	Strongly positive	9				1
	ND	3	19			1
**Aberrant IELs** (%)	Mean (SD;range)	-	-	4,0	64	7
				(6.0; 2-19)	(23; 20-96)	(32; 1-87)
**EATL type**	Primary	-	-	-	-	4
	Secondary					8

*ACD*: active coeliac disease patients; *GFD*: coeliac disease patients on a gluten-free diet; *RCDI and II*: refractory coeliac disease type I and II; *EATL*: enteropathy associated T-cell lymphoma; *EMA*-*A*: endomysial antibodies; *tTGA*-*A*: tissue transglutaminase antibodies; *IELs*: intra-epithelial lymphocytes.

### Active CD versus GFD

Serum levels of the inflammatory chemokine IL-8 (p = 0.00) and the T-cell activation factor sCD25 were higher in active CD patients than in patients in remission on a GFD (p = 0.00, highly significant) as well as the Th-17 lineage-defined cytokine IL-17 levels (p = 0.011, moderately significant). Serum levels of IL-6, IL-22, sCD27, sMICA, granzyme-B and sCTLA-4 were not significantly elevated in the ACD group. In addition, levels of CRP, ESR, albumin and leukocyte counts were similar (data not shown).

### Uncomplicated CD versus RCD

The serum levels of IL-8, IL-17 and sCD25 in RCD type I and type II were comparable to those in the ACD group, however, significantly lower levels were observed in the GFD group. Moreover, in comparison to both ACD and GFD patients, RCDII patients showed increased levels of granzyme-B (p = 0.020; p = 0.016, both moderately significant) and IL-6 (p = 0.018; p = 0.024, both moderately significant), respectively. Furthermore, serum levels of soluble CTLA-4 were lower in RCDII patients than those in remission upon a GFD (p = 0.003, moderately significant). Similar IL-22 serum levels were found in uncomplicated CD and RCDI-II. .

**Figure 2:**
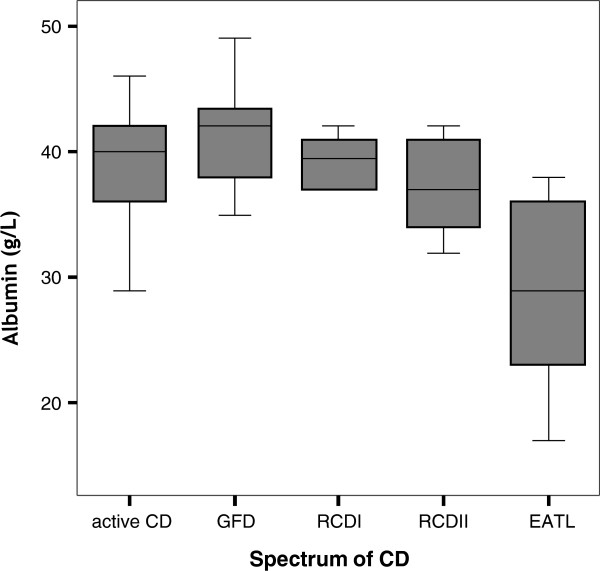
**Concentration of albumin in the different subsets of the CD spectrum. **The concentration of albumin is significantly higher in both the ACD (p = 0.003) and the GFD (p = 0.000) subset compared to the complicated forms of CD. In EATL patients clearly lower concentrations compared to both RCDI and II (p = 0.003 and p = 0.037, respectively) were observed. Abbreviations: CD, coeliac disease; ACD, active coeliac disease; GFD, response upon a gluten-free diet; RCD I, refractory coeliac disease type I; RCD II, refractory coeliac disease type II; EATL, enteropathy associated T-cell lymphoma.

Comparison of the inflammatory parameters CRP, ESR and leukocyte count did not reveal significant differences between uncomplicated CD and RCDI-II. The concentration of albumin was lower in both RCD subsets when compared to ACD (p = 0.003, moderately significant) and GFD (p = 0.000, highly significant; Figure 2).

### The RCD complex

None of the markers tested was able to distinguish RCDII from RCDI. Moreover, no significant differences in levels of albumin or inflammatory parameters CRP, ESR and leukocyte count were observed.

### EATL versus ACD and RCD

The highest serum levels of IL-6 were observed in the EATL group, were higher (p = 0.000, highly significant) as compared to the ACD and GFD groups, as well as higher (p = 0.002; p = 0.007, moderately significant) than RCDI-II, respectively. Moreover, serum levels of IL-6 were clearly elevated in EATL over RCDII with an AUC of 0.82 [95% CI: 0.649-0.971]. IL-6 levels in EATL patients tended to correlate (0.45, p = 0.08) with CRP levels, but not with IL-17 levels. Furthermore, serum levels of sCD27 were decreased (moderately significant) in EATL patients as compared to all other groups, except RCDII in which (highly) significant (p = 0.000) differences were found). Nevertheless, ROC analysis resulted in a very low AUC. In addition, similar levels of sMICA, granzyme-B and sCD25 were measured in EATL and RCDI-II. However, the serum albumin concentration in EATL patients was lower (moderately significant) than in ACD and both types of RCD (Figure 2).

## Discussion

Currently, physicians are unable to predict who will develop complicated CD. As invasive techniques are still required to differentiate between uncomplicated and complicated forms of CD, the purpose of the present analysis was to evaluate the ability of markers in peripheral blood to distinguish the various CD subsets. By doing so, it may also provide insight in the potential differences in underlying immunopathomechanisms of these disease subsets.

The results could be biased by cytokine production from different cellular sources in the peripheral blood, and therefore may not exactly mimic the intestinal inflammation [20]. However, other investigators have previously shown that blood cytokine profiles do reflect intestinal mRNA expression in active CD patients [2,21-23]. Care must be taken in case our findings are extrapolated to other age groups, as it cannot be excluded that normal values vary over age.

### Active CD versus GFD

Our results are in keeping with previous studies reporting on serum cytokine levels in ACD patients, that showed up-regulation of IL-8 [20,24], a chemokine produced by macrophages, epithelial as well as endothelial cells that attracts leukocytes to a site of inflammation. The same accounts for the observed elevated levels of sCD25, that is cleaved off from the membranous CD25 (IL-2R-alpha) during T-cell activation [25-27]. Furthermore, the increased levels of the pro-inflammatory cytokine IL-17 observed in the current study, supports the view that Th17 cells that produce this particular cytokine are involved in several auto-immune diseases and are suggested to have a pathogenic role in CD [3-5]. In contrast to the (pro-) inflammatory environmental characteristics observed in this analysis and to previous reports [20,26,28], the levels of IL-6, illustrative of an acute phase response and a potent inducer of the Th17 pathway [29], were low and similar in both uncomplicated CD subsets. A clear explanation for these findings is lacking, especially as strongly increased levels were found in RCDII and EATL patients. Another cytokine involved in gut inflammation is IL-22 that is produced by Th17 and/or Th22 cells, where it is currently believed to exert regulatory functions [30,31]. Elevated levels of IL-22 have been found in the mucosa of Crohn’s disease [32], but its role in CD is yet unclear [3,33]. Nevertheless, our results failed to reveal a correlation with IL-22 serum levels and disease activity or disease entity. sCD27 serum levels are increased in T-cell mediated diseases, including some auto-immune disorders [34,35]. However, the sCD27 levels were normal in ACD. This in contrast to the previously reported increased production of another lymphocyte activation marker sCD25. This dissimilarity is remarkable as in SLE patients sCD25 and sCD27 levels are strongly correlated during the whole disease course [36].

### The RCD complex

Although, by general consent, RCD type I and II are considered two related disease entities within the spectrum of CD, it remains unclear if both diseases share a similar pathogenesis responsible for the gluten-independent inflammation. In keeping with the current opinion, our results showed in both types of RCD similar inflammatory characteristics and T-cell activation based on serum levels of IL-8, IL-17, IL-22 and sCD25, respectively. On the other hand, a transition from RCDI to RCDII has only been reported sporadically [16]. Theoretically, granzyme-B is a suited parameter to differentiate between RCD type I and II, since aberrant IELs are clearly cytotoxic and express high amounts of intracellular granzyme-B [12]. Although increased levels of soluble granzyme-B were observed in RCDII patients, these were not significantly higher than in RCDI patients.

### RCD versus uncomplicated CD

Currently, it is unknown to what extent the gluten-independent inflammation as generally observed in RCD evolved from and/or differs from the gluten induced inflammation in active CD. In this study, the pro-inflammatory T-cell response, including IL-8, IL-17, sCD25, in both types of RCD and ACD patients shows resemblance, with exception of evidently increased IL-6 levels in RCDII over active CD. This finding suggest a higher inflammatory state in RCDII than in ACD, however, similar levels of the inflammatory parameters CRP, ESR and leukocyte count were observed. In line with the lack of intestinal inflammation in patients adhering to a GFD, the pro-inflammatory response was significantly lower as compared to the complicated forms of CD. Interestingly, in comparison to ACD patients, RCDII patients displayed a distinctive cytotoxic T-cell activation profile based on elevated serum levels of granzyme-B. The levels of these parameters observed in RCDI patients did not differ from either ACD or RCDII patients.

### Monitoring EATL development

RCDII patients carry a high risk to develop an EATL, yet, so far no serum parameters for EATL development have been identified, including efforts in the present analysis. Based on the fact that EATL cells contain large amounts of granzyme-B [13], these levels were measured in the peripheral blood, but EATL patients did not contain higher levels than active CD or RCD patients. Furthermore, elevated levels of sCD27 [37] and sCD25 [38] have been suggested to be associated with tumour burden in some lymphoid neoplasia. Neoplastic lymphoid cells in non-Hodgkin lymphoma express CD27 and are considered responsible for the increased sCD27 production [37]. The current analysis failed to show increased sCD27 levels in EATL patients and thereby suggests that EATL regards a distinct type of lymphoma and does not aid in the identification of this particular lymphoma. In contrast to a previous study showing elevated sCD25 levels in EATL [39], we found comparable levels of sCD25 in EATL, RCDI-II and ACD. The same accounts for sMICA, that is cleaved from membranous MICA and has been shown to impair NKG2D mediated tumor surveillance in epithelial tumors [40], as well as it has shown potential as a prognostic parameter in hematopoietic malignancies since increased levels of sMICA were found in leukemia patients [41]. The only marker that distinguished EATL from all other groups was IL-6 and its levels correlated with CRP levels, indicating a more severe acute inflammatory response in EATL patients that is distinctive from the other subsets of CD. In accordance with our data, an association with IL-6 levels and survival in Hodgkin lymphoma has been recognized almost twenty years ago [42].

### Cytokine levels in comparison with other gastro-intestinal diseases

Our data suggests that complicated CD is accompanied by a higher pro-inflammatory state as compared to uncomplicated CD. To provide insight in the extend of this inflammation, it can b compared to the cytokine profile in other (small) intestinal disease, such as Crohn’s disease. Not only have elevated serum levels of IL-6 been reported in this disease, but these levels appear even higher than in complicated CD [43]. On the other hand, IL-6 and IL-8 serum levels in *Helicobacter Pylori* infected patients with peptic ulcer disease are not elevated [44].

### Clinical implication and potential application

Taken together, for daily clinical practice the results of this analysis suggest that apart from detection of CD associated antibodies and duodenal biopsies, other variables including IL-8, sCD25 and possibly IL-17, might be helpful in monitoring inflammatory disease status and differentiating between patients on a strict GFD and those diagnosed as having RCDI-II. In case CD specific antibodies remain mildly elevated, which is not rare in RCDI-II patients, serum levels of granzyme-B could possibly serve as an additional markers to distinguish ACD from RCDII, and will enable early intervention. However, the currently accepted diagnostic work-up of RCD remains required, pending prospective serum studies including larger series of patients.

## Conclusion

In conclusion, both types of RCD are characterised by an ongoing and/or recurring inflammatory disease status showing great resemblance to that observed in ACD despite strict adherence to a GFD, yet differentiates itself by elevated serum IL-6 concentrations in RCDII. Furthermore, in addition to this increased pro-inflammatory profile, RCDII reveals a distinctive cytotoxic T-cell activation profile as compared to ACD based on elevated levels of granzyme-B, whereas RCDI does not. Although no EATL-specific or -associated immunological parameters were found in this study, our ongoing efforts may identify relevant markers. Further research will also address the prospective and diagnostic value of the serum variables identified is this study in order to expand the clinical application of (R)CD serology beyond standard autoantibody testing.

## Abbreviations

ACD: Active coeliac disease; CD: Coeliac disease; IEL: Intraepithelial lymphocyte; GFD: Gluten-free diet; RCD: Refractory coeliac disease; EATL: Enteropathy associated T-cell lymphoma.

## Competing interests

Unrestricted grant from Astra Zeneca. Supported by the Coeliac Disease Consortium, The Netherlands. There is no competing interest.

## Authors’ contributions

GT conceived of the study, contributed to acquisition and interpretation of data and drafted the manuscript. RW carried out the ELISA and drafted the manuscript. BB and MS conceived of the study, and participated in its design and coordination and helped to draft the manuscript. HA and PB carried out the ELISA. VC performed the statistical analysis. CM participated in its design and coordination and helped to draft the manuscript. All authors read and approved the final manuscript.

## Pre-publication history

The pre-publication history for this paper can be accessed here:

http://www.biomedcentral.com/1471-230X/12/159/prepub
